# Supplementary material for: Association between thyroid hormone sensitivity and ischemic stroke-associated pneumonia: The role of FT3/FT4 ratio
Source: PLoS One. 2025 Nov 26;20(11):e0333057. doi: 10.1371/journal.pone.0333057 (PMC12654940; doi:10.1371/journal.pone.0333057)
Supplement: S1 Table — Supplementary Table 1 presents the changes in standardized mean differences (SMDs) for baseline covariates before and after 1:1 matching between the iSAP (n = 376) and non-iSAP (n = 1391) groups using a combination of propensity score matching (PSM) and Genetic Matching (GenMatch) algorithms. After matching, both groups comprised 1463 patients each. Most covariates showed a marked reduction in SMDs, with values falling below 0.1, indicating satisfactory baseline balance. Abbreviations are as defined in Table 1. Additional abbreviations: SMD, Standardized Mean Difference; PSM, Propensity Score Matching; GenMatch, Genetic Matching. (DOCX) [file pone.0333057.s001.docx]

**Supplementary Table 1. Comparison of Standardized Mean Differences (SMDs) of Covariates Before and After Propensity Score Matching (PSM) Combined with Genetic Matching (GenMatch)**

| Covariates | Standardized Difference  (Before Matching) | Standardized Difference  (After Matching) |
| --- | --- | --- |
| Age | 0.55 (0.44, 0.67) | 0.1675 |
| Sex | 0.09 (-0.03, 0.20) | 0.0252 |
| Current smoking | 0.00 (-0.11, 0.11) | 0.0237 |
| Hypertension | 0.02 (-0.10, 0.13) | 0.0483 |
| Diabetes | 0.02 (-0.10, 0.13) | 0.1533 |
| Atrial fibrillation | 0.58 (0.47, 0.70) | 0.0036 |
| COPD | 0.40 (0.28, 0.51) | 0.0379 |
| FPG | 0.22 (0.10, 0.33) | 0.0265 |
| TG | 0.37 (0.26, 0.49) | 0.1116 |
| HbA1C | 0.04 (-0.08, 0.16) | 0.0672 |
| TC | 0.11 (-0.00, 0.23) | 0.0619 |
| HDL-C | 0.13 (0.02, 0.25) | 0.1301 |
| LDL-C | 0.07 (-0.04, 0.19) | 0.0370 |
| CRP | 0.76 (0.64, 0.88) | 0.0942 |
| AST | 0.33 (0.21, 0.44) | 0.0380 |
| ALT | 0.06 (-0.05, 0.18) | 0.0498 |
| BUN | 0.39 (0.28, 0.51) | 0.0024 |
| UA | 0.09 (-0.02, 0.21) | 0.0319 |
| WBC | 1.00 (0.89, 1.12) | 0.2106 |
| HCY | 0.15 (0.03, 0.26) | 0.0017 |
| EGFR | 0.21 (0.10, 0.33) | 0.0204 |
| NHISS | 0.74 (0.62, 0.86) | 0.0259 |
| GCS | 0.83 (0.71, 0.94) | 0.0681 |

Supplementary Table 1 presents the changes in standardized mean differences (SMDs) for baseline covariates before and after 1:1 matching between the iSAP (n=376) and non-iSAP (n=1391) groups using a combination of propensity score matching (PSM) and Genetic Matching (GenMatch) algorithms. After matching, both groups comprised 1463 patients each. Most covariates showed a marked reduction in SMDs, with values falling below 0.1, indicating satisfactory baseline balance.

Abbreviations: SMD, Standardized Mean Difference; COPD, Chronic Obstructive Pulmonary Disease; FPG, Fasting Plasma Glucose; TG, Triglycerides; HbA1C, Glycated Hemoglobin A1c; TC, Total Cholesterol; HDL-C, High-Density Lipoprotein Cholesterol; LDL-C, Low-Density Lipoprotein Cholesterol; CRP, C-Reactive Protein; AST, Aspartate Aminotransferase; ALT, Alanine Aminotransferase; BUN, Blood Urea Nitrogen; UA, Uric Acid; WBC, White Blood Cell Count; HCY, Homocysteine; eGFR, Estimated Glomerular Filtration Rate; NIHSS, National Institutes of Health Stroke Scale; GCS, Glasgow Coma Scale.
