# Supplementary material for: Association between thyroid hormone sensitivity and ischemic stroke-associated pneumonia: The role of FT3/FT4 ratio
Source: PLoS One. 2025 Nov 26;20(11):e0333057. doi: 10.1371/journal.pone.0333057 (PMC12654940; doi:10.1371/journal.pone.0333057)
Supplement: S1 Fig — This figure displays the standardized mean differences (SMDs) for baseline covariates between the ischemic stroke-associated pneumonia (iSAP) group and the non-iSAP group before and after propensity score matching (PSM) using the Genetic Matching algorithm. The horizontal axis represents the absolute value of SMDs, with a commonly accepted threshold of 0.1 (dashed vertical line) indicating adequate balance. Covariate balance substantially improved after matching, as evidenced by most post-matching SMDs falling below the 0.1 threshold. This demonstrates that the matched sample achieved a high degree of comparability between groups, thereby minimizing potential confounding bias in subsequent analyses. (DOCX) [file pone.0333057.s002.docx]

**Supplementary Figure 1. Standardized Mean Differences of Baseline Covariates Before and After Propensity Score Matching with Genetic Matching Algorithm.**

**Legend:** This figure displays the standardized mean differences (SMDs) for baseline covariates between the ischemic stroke-associated pneumonia (iSAP) group and the non-iSAP group before and after propensity score matching (PSM) using the Genetic Matching algorithm. The horizontal axis represents the absolute value of SMDs, with a commonly accepted threshold of 0.1 (dashed vertical line) indicating adequate balance. Covariate balance substantially improved after matching, as evidenced by most post-matching SMDs falling below the 0.1 threshold. This demonstrates that the matched sample achieved a high degree of comparability between groups, thereby minimizing potential confounding bias in subsequent analyses.
