# Supplementary material for: Association between thyroid hormone sensitivity and ischemic stroke-associated pneumonia: The role of FT3/FT4 ratio
Source: PLoS One. 2025 Nov 26;20(11):e0333057. doi: 10.1371/journal.pone.0333057 (PMC12654940; doi:10.1371/journal.pone.0333057)
Supplement: S1 Abbreviations — (DOCX) [file pone.0333057.s003.docx]

**List of abbreviations**

iSAP Ischemic Stroke-Associated Pneumonia

AIS Acute Ischemic Stroke

FT3/FT4 Ratio of Free Triiodothyronine to Free Thyroxine

FT3 Free Triiodothyronine

FT4 Free Thyroxine

TSHI Thyroid-Stimulating Hormone Index

TT4RI Thyrotroph T4 Resistance Index

TFQI Thyroid Feedback Quantile-based Index

TFQI-FT3 Thyroid Feedback Quantile-based Index calculated with FT3

TFQI-FT4 Thyroid Feedback Quantile-based Index calculated with FT4

ROC Receiver Operating Characteristic

AF Atrial Fibrillation

COPD Chronic Obstructive Pulmonary Disease

NIHSS National Institutes of Health Stroke Scale

OR Odds Ratio

A_2_DS_2_ Age, Atrial Fibrillation, Dysphagia, Sex, and Stroke Severity Score

AUC Area Under the Curve

MRI Magnetic Resonance Imaging

KWDT Kubota Water Drinking Test

GCS Glasgow Coma Scale

WBC White Blood Cell count

CRP C-Reactive Protein

FPG Fasting Plasma Glucose

AST Aspartate Aminotransferase

ALT Alanine Aminotransferase

HbA1c Glycated Hemoglobin A1c

HCY Homocysteine

SCr Serum Creatinine

ALB Albumin

TG Triglycerides

TC Total Cholesterol

HDL-C High-Density Lipoprotein Cholesterol

LDL-C Low-Density Lipoprotein Cholesterol

TSH Thyroid-Stimulating Hormone

eGFR Estimated Glomerular Filtration Rate

SAP Stroke-Associated Pneumonia

PaO₂ Partial Pressure of Arterial Oxygen

FiO₂ Fraction of Inspired Oxygen

TH Thyroid Hormone

SD Standard Deviation

IQRs Interquartile Ranges

BUN Blood Urea Nitrogen

UA Uric Acid

CIs Confidence Intervals

NRI Net Reclassification Improvement

IDI Integrated Discrimination Improvement

NTIS Non-Thyroidal Illness Syndrome

ATA American Thyroid Association

WHO World Health Organization

RCTs Randomized Controlled Trials
