# Supplementary material for: Association between thyroid hormone sensitivity and ischemic stroke-associated pneumonia: The role of FT3/FT4 ratio
Source: PLoS One. 2025 Nov 26;20(11):e0333057. doi: 10.1371/journal.pone.0333057 (PMC12654940; doi:10.1371/journal.pone.0333057)
Supplement: Attachment — Submitted filename: Response to Reviewers.docx [file pone.0333057.s006.docx]

Dear Editor and Reviewers,

We sincerely thank the editor and reviewers for their thoughtful and thorough evaluation of our manuscript entitled “**Association Between Thyroid Hormone Sensitivity and Ischemic Stroke‑Associated Pneumonia: The Role of FT3/FT4 Ratio**” (Manuscript ID: **PONE‑D‑25‑07372R1**). Their constructive comments greatly strengthened the manuscript’s clarity, methodological rigor, and overall quality.

In response, we carefully revised the manuscript and prepared detailed point‑by‑point responses. Key revisions include clarifying methodological procedures (e.g., matching methods and biomarker measurements), improving terminology and OR interpretation, adding supplementary data (e.g., balance diagnostics and updated tables), and expanding the discussion of study limitations. These revisions do not alter the study’s primary conclusions. They instead enhance its transparency and interpretability.

Below, we provide detailed responses to each comment from the reviewers, with corresponding revisions indicated in the manuscript (line numbers refer to the revised version).

**Reviewer #2 – General Comment**

We thank Reviewer #2 for raising detailed methodological points, including sample size imbalance, eGFR calculation and reference ranges, biomarker sample type, FT3/FT4 ratio reporting in Table 1, and standardization of testing methods. We have carefully revised the manuscript to address each of these points in detail, as summarized below.

**Reviewer #2 Comment** **(Original)**

The paper discusses an important point regarding thyroid hormones in stroke pateints, but the methods used to address the problem need to be improved, for example the difference between the control and study group number is large (iSAP group (n = 376) and the control group (n = 1,391). The eGFR values need to be repeated, what are the normal ranges of eGFR? Did you use serum or plasma for biomarker measurements? The ratio of thyroid hormones FT3/FT4 in table 1 is omitted? the tests used need to be standard or proven by an international society or committe.

**Response:**

Your comments highlighted important methodological and statistical issues requiring clarification, which led us to refine these sections. Please see our point-by-point responses below:

**1. Large difference in sample sizes between the non‑iSAP and iSAP groups**

**Response:** This comment drew our attention to the sample size imbalance between groups and prompted us to address it by combining propensity score matching (PSM) with Genetic Matching (GenMatch) to minimize baseline covariate imbalance and improve comparability.

**Revision details:** Matching was performed in a 1:1 ratio using nearest‑neighbor methods with replacement. This allowed certain iSAP patients to be matched multiple times to achieve optimal covariate balance and is widely used in observational studies when group sizes differ substantially.

**New results:** The final matched cohort included 2,926 patients (1,463 in each group). Supplementary Table 1 and Supplementary Figure 1 show that the absolute standardized mean differences (SMDs) for most covariates were reduced to below 0.1, indicating excellent matching quality.

**Location in manuscript:** Additional methodological details are provided in the revised **Methods (Lines 199–208),** and the balance analysis results are now presented in the **Results (Lines 257–266).**

Following matching, we performed multivariate logistic regression in the matched cohort. The results are shown in **Table 4 (Line 376)**. The FT3/FT4 ratio was significantly associated with iSAP risk (Q2 vs. Q1: OR = 0.69, 95% CI: 0.56–0.86, p = 0.0011; Q4 vs. Q1: OR = 0.72, 95% CI: 0.57–0.91, p = 0.0054). TT4RI and TFQI‑FT4 showed positive associations, while TFQI‑FT3 remained significant in the fully adjusted model **(Results, Lines 353–357).**

We noted that TSHI and TFQI-FT3 exhibited attenuated or reversed associations after matching. This likely reflects:

(1) Improved balance in sample structure reducing residual confounding;

(2) Nonlinear interactions between TFQI indices and unmatched variables manifesting as directional shifts in extreme quartiles, a common phenomenon in observational studies.

**2. Calculation method and reference range for eGFR**

**Response:** We clarified in the revised manuscript that **eGFR was calculated from serum creatinine (Scr) using the CKD-EPI formula.**

**Location in manuscript:** Methods (Lines 145–146).

**Reference range:** The normal range is 90–120 mL/min/1.73 m².

**3. Sample type used for biomarker measurements**

**Response:** We clarified in the revised manuscript that serum creatinine was measured using serum samples.

**Location in manuscript:** Methods (Lines 139–140).

**4. Omission of FT3/FT4 ratio in Table 1**

**Response:** We corrected the omission and added FT3/FT4 ratio values for the control group to ensure completeness of baseline characteristics:

non-iSAP group: 0.31 (0.26–0.38)

iSAP group: 0.25 (0.21–0.30)

**Location in manuscript:** Revised Table 1 (Line 267).

**5. Standardization of testing methods**

**Response:** We clarified that all thyroid hormone indices were measured using electrochemiluminescence immunoassay (ECLIA) on the Roche Cobas analyzer, a method widely endorsed by international endocrine societies.

**Location in manuscript:** Methods (Lines 142–144).

**Summary for Reviewer #2:**

These revisions comprehensively address the reviewer’s concerns regarding methodological rigor and data clarity, while preserving the overall conclusions of the study.

**Reviewer #4 – General Comment**

We thank Reviewer #4 for insightful feedback highlighting key areas for improvement, such as OR interpretation, study design terminology, potential misclassification and COPD‑related confounding, data provenance, and minor language issues.

**Reviewer #4 Comment (Original)**

It appears that a number of reviewers have already and thoroughly reviewed this manuscript, thus, I am not going to repeat what most of them observed/commented on, particularly Reviewer 1 whose review was thorough and insightful. I am only commenting on points that were not raised by other reviewers, or those which I think the authors’ response was not satisfactory.

**Comment 1: Odds ratio (OR) interpretation and wording**

Reviewer 1 had issues interpreting ORs, to which the authors responded by modifying the wording. I still think the wording is confusing: for example, in the abstract the authors wrote:

- “Univariate analysis demonstrated that lower FT3 levels (OR = 0.85, 95% CI:28 0.80-0.91, p< 0.0001) and a reduced FT3/FT4 ratio (OR = 0.80, 95% CI: 0.69-0.92, p29= 0.0018) were significantly associated with an increased risk of iSAP.” And then in the next sentence they wrote:

- “After adjusting for confounders, multivariate analysis revealed that a higher FT3/FT4 ratio was independently associated with a lower iSAP risk (Q3 vs. Q1: OR= 0.40, 95% CI:32 0.26-0.62, p < 0.0001; Q4 vs. Q1: OR = 0.31, 95% CI: 0.19-0.48, p < 0.0001).”

This is confusing: the first sentence implies the lower FT3 had OR<1 which was interpreted as “increased risk of iSAP”(?!), but the second sentence implies that higher FT3 (Q3 vs Q1 and Q4 vs Q1) were associated with, again, OR<1, but this time interpreted as “lower iSAP risk”. Later in main text, lines 252-255, the authors wrote: “Regarding thyroid hormone parameters, lower FT3 levels were associated with increased odds of developing iSAP (OR = 0.85, 95% CI: 0.80–253 0.91, p < 0.0001), as was a lower FT3/FT4 ratio (OR = 0.80, 95% CI: 0.69–0.92, p =254 0.0018)”. Similar to the first sentence in abstract above, this is incorrect: An increased odds of iSAP cannot be an OR of 0.85; This is an error in wording, not a counter-intuitive finding as the author suggested in one of their responses to reviewers’ comments. Results of univariate and multivariate analyses should be consistent in terms of what the ORs represent. As Reviewer 1 commented, an OR of <1 is generally perceived as lower risk, which is indeed reflected in the second sentence above in the authors’ own wording. I suggest the wording to be corrected in the first sentence, and elsewhere as needed, so that the reader is clear about the interpretation of these results. Alternatively, the authors could have considered re-defining the reference group for T3 levels, so that Q3 (the highest level) is the reference, and then compare to it Q2 and Q1 (lowest levels); had this been done, the OR would have reflected the increased risk in iSAP, with OR >1, which is more intuitive. In my view, the wording in the second sentence above is clear and correct, so I don’t see a need to re-do the analysis. Indeed, “higher T3 associated with lower risk of iSAP” is the equivalent (flip side if you well) of lower T3 associated with higher risk of iSAP.

**Response:**

We appreciate the reviewer’s observation regarding this inconsistency. After reviewing both the abstract and results sections, we recognized that our previous wording may have caused confusion for readers. We have revised all relevant sentences so that OR < 1 is consistently interpreted as indicating a protective association. Specifically:

In the **Abstract (Lines 28–30),** we now describe higher FT3 levels and higher FT3/FT4 ratio as being associated with lower iSAP risk.

In the **Results section (Lines 325–327),** similar adjustments were made to maintain consistency.

These changes ensure that the statistical interpretation is accurate and presented in a way that is straightforward for readers to follow.

**Comment 2: Use of “case-control” terminology**

I think the use of case-control design-suggestive wording, such as referring to “cases” as those with iSAP and comparing then to “controls” who had no iSAP. This does not align with conventional epidemiologic wisdom. Since all participants had AIS, and the comparison is between those who did and did not develop iSAP, the analysis is essentially cross-sectional within a retrospective AIS cohort, as exposure was measured at a single point that preceded iSAP. By conditioning on AIS, the authors already restricted their base population and precludes conducting a methodologically sound case-control study. I therefore do not think the use of “controls” is sound in this manuscript. Referring, or even suggesting, to this as a case-control study risks confusion about control selection and the interpretation of odds ratios.

**Response:**

We appreciate the reviewer’s concern about potential confusion from case‑control terminology. To prevent this, we replaced the term “control” with “non‑iSAP” throughout the manuscript and tables and clarified this change in the Methods section.

In the **Methods (Lines 96–100)**, we clarified that thyroid hormone indices were measured at admission and iSAP was assessed during hospitalization, underscoring the temporal relationship.

We also explicitly describe the study as a **retrospective cohort** rather than case-control design.

**Comment 3: Potential misclassification of iSAP and baseline infection status / COPD confounding / hypothesis-generating framing**

How did the authors establish the criterion “Patients who had infectious diseases or fever within 2 weeks prior to admission and those who used antibiotics within 1 week before admission were excluded”? Do they have access to regional/state level health records, or just relied on their own centre’s records? I am concerned the CRP level in the iSAP group is near to 7 (seven) times that of the non-iSAP group, this is shortly after admission so I am wondering whether the former group was truly infection/pneumonia-free upon admission. The markedly elevated CRP levels observed at admission in the iSAP group (median 13.6 vs. 2.0 mg/L) raise concerns about potential misclassification of the outcome. It is plausible that some patients labeled as developing iSAP during hospitalization were already infected at admission, which would render the temporal relationship between thyroid hormone levels and infection ambiguous. The significantly higher prevalence of COPD in the iSAP group (15.7% vs. 4.0%) further supports the concern that these patients were at a fundamentally different baseline risk for infection and inflammation. While COPD was included as a covariate in the fully adjusted model, this binary adjustment does not adequately account for disease severity or its systemic inflammatory impact. More importantly, statistical adjustment cannot resolve the misclassification of prevalent pneumonia as incident iSAP, nor can it correct for potential reverse causality (i.e., low FT3 as a marker of acute illness rather than a causal factor). These limitations undermine the validity of the observed associations and should be acknowledged explicitly in the discussion. More importantly, rather than implying a protective or harmful effect, the observed association between FT3 and the odds of iSAP should be interpreted as hypothesis-generating, pending confirmation in longitudinal studies, as Reviewer 1 recommended.

Reviewer Comment Part 1: Potential misclassification of iSAP and baseline infection status

“How did the authors establish the criterion… the markedly elevated CRP levels… raise concerns about potential misclassification…”

**Response:**

We implemented three specific measures to address these concerns:

**1. Misclassification of iSAP**

The exclusion of pre-existing infections relied on our hospital’s integrated EMR, which includes inpatient/outpatient data and provincial-level visit records. This system enables clinicians to review prior diagnoses and treatments.

We added a description in the **Methods (Lines 91–96)** clarifying this process and, in the **Definitions section** **(Lines 149–152)**, specified that all patients underwent chest CT and infection screening at admission to ensure iSAP was hospital-acquired.

SAP diagnoses followed the Pneumonia in Stroke Consensus Group criteria and were independently confirmed by two neurologists, and respiratory specialists were consulted when necessary.

**2. COPD confounding**

We clarified in the **Methods (Lines 128–130)** that COPD diagnoses were based on confirmed medical records, rather than patient self‑reports.

In the **Discussion – Limitations (Lines 535–539)**, we acknowledged residual confounding due to lack of COPD severity data.

**3. Hypothesis-generating framing**

We revised language across the manuscript to avoid implying causality.

In the **Discussion – Limitations (Lines 556–561),** we added:

“However, given the retrospective design, the observed associations between thyroid hormone sensitivity indices and iSAP should be interpreted as hypothesis-generating rather than implying causality. In particular, the inverse relationship between FT3 levels and iSAP risk may reflect a marker of disease severity rather than a causal factor. Therefore, longitudinal cohort studies are warranted to verify the temporal and mechanistic links.”

Similar wording changes were applied to the **Abstract (Lines 30, 32, 34–35, 40–43),** **Discussion (Lines 425–427, 440–442, 471–472, 495–497)** and **Conclusion (Lines 563–568)** for consistency.

**Comment 4: Data provenance and potential duplication**

The authors wrote in their methods “The data for this study were derived from a previously established retrospective cohort study on the prognosis of ischemic stroke.” But they do not cite any studies. This is important to clarify whether the same results were published previously, in full or in part, and to clarify the added value of this submitted update.

**Response:**

The data originate from a previously established stroke cohort, while the present analysis is novel, focusing on thyroid hormone sensitivity indices (FT3/FT4, TT4RI, TSHI, TFQI) and related hypotheses that have not been previously reported. The statistical methods used in this work are also original to this study, and we clarified this information in the **Methods (Lines 81–86)**.

**Comment 5: Language and typographical issues**

Most other reviewers commented on the need for language proofing, yet, still I see issues remaining. For example, line 114: “(AF)and “: missing space.

**Response:**

We have conducted a thorough proofreading of the entire manuscript and corrected all typographical and formatting issues. This includes the missing space at line 114.

**Summary for Reviewer #4**

Collectively, these revisions clarify OR interpretation, refine study terminology, address potential misclassification and COPD confounding, and improve language quality and data provenance, enhancing the manuscript’s methodological soundness and readability.
